# Transmembrane and Coiled-Coil Domain Family 1 Is a Novel Protein of the Endoplasmic Reticulum

**DOI:** 10.1371/journal.pone.0085206

**Published:** 2014-01-14

**Authors:** Chao Zhang, Yik-Shing Kho, Zhe Wang, Yan Ting Chiang, Gary K. H. Ng, Pang-Chui Shaw, Yuzhuo Wang, Robert Z. Qi

**Affiliations:** 1 Division of Life Science and State Key Laboratory of Molecular Neuroscience, The Hong Kong University of Science and Technology, Clear Water Bay, Kowloon, Hong Kong, China; 2 Department of Experimental Therapeutics, British Columbia Cancer Agency, Vancouver, British Columbia, Canada; 3 Biochemistry Programme and Centre for Protein Science and Crystallography, School of Life Sciences, The Chinese University of Hong Kong, Shatin, New Territories, Hong Kong, China; Ecole Polytechnique Federale de Lausanne, Switzerland

## Abstract

The endoplasmic reticulum (ER) is a continuous membrane network in eukaryotic cells comprising the nuclear envelope, the rough ER, and the smooth ER. The ER has multiple critical functions and a characteristic structure. In this study, we identified a new protein of the ER, TMCC1 (transmembrane and coiled-coil domain family 1). The TMCC family consists of at least 3 putative proteins (TMCC1–3) that are conserved from nematode to human. We show that TMCC1 is an ER protein that is expressed in diverse human cell lines. TMCC1 contains 2 adjacent transmembrane domains near the C-terminus, in addition to coiled-coil domains. TMCC1 was targeted to the rough ER through the transmembrane domains, whereas the N-terminal region and C-terminal tail of TMCC1 were found to reside in the cytoplasm. Moreover, the cytosolic region of TMCC1 formed homo- or hetero-dimers or oligomers with other TMCC proteins and interacted with ribosomal proteins. Notably, overexpression of TMCC1 or its transmembrane domains caused defects in ER morphology. Our results suggest roles of TMCC1 in ER organization.

## Introduction

The endoplasmic reticulum (ER) is a continuous network of membranes in eukaryotic cells that extends throughout the cytoplasm. The functions of the ER, one of the largest organelles in cells, have been studied extensively, including the translocation of proteins across the ER membrane [1], [2], the folding of proteins in the ER lumen [3], [4], the transport of proteins from ER to the Golgi apparatus [5], [6], the synthesis of lipids and steroids [7], [8], and the regulation of cellular Ca^2+^ concentrations [9], [10]. The ER is composed of the nuclear envelope and the peripheral ER. The nuclear envelope, which has a double lipid bilayer structure, surrounds the nucleus and connects to the peripheral ER. Electron microscopy has shown that the peripheral ER can be classified based on morphology into rough ER and smooth ER, which perform distinct functions in cells. Rough ER, defined by the presence of membrane-bound ribosomes, is responsible for the translation, translocation, and folding of membrane and secretory proteins. By contrast, smooth ER, defined by the absence of membrane-bound ribosomes, is required for lipid synthesis, steroid metabolism, and regulation of Ca^2+^ concentrations in cells.

The ER has a characteristic shape that is evolutionarily conserved. Based on membrane curvature, the ER structure can be divided into 2 distinct morphological domains: sheets and tubules [11]–[13]. ER sheets, with little membrane curvature, contain flat membranes and form ER cisternae. By contrast, ER tubules, which show highly curved membranes in cross-section, have a polygonal pattern connected by 3-way junctions [13]. ER sheets and tubules correspond generally to the rough and smooth ER, respectively. Moreover, because nuclei are large organelles, the spherical nuclear envelope is also considered a flat ER sheet [11]. The functions of ER are known to be related closely to the ER’s structural features, but the mechanisms that generate and maintain the distinct ER morphologies are not understood fully. A few key proteins, however, have been found to play critical roles in regulating the ER’s morphology.

First, 2 families of integral membrane proteins have been identified as being responsible for the formation of ER tubules: reticulons and DP1/Yop1p [14]. In yeast and mammalian cells, these proteins localize in ER tubules and are excluded from ER sheets. Overexpression of certain reticulon proteins leads to the assembly of long and unbranched tubules, whereas the absence of both reticulons and Yop1p in yeast leads to the loss of tubular ER [14]. Moreover, purified proteins of these 2 families are sufficient for deforming reconstituted yeast proteoliposomes into tubules [15]. Proteins of these families contain a domain with 2 long hydrophobic fragments that form hairpins within the ER membrane. These hairpins may form wedges in the membrane to generate the high curvature observed in cross-section [14]. The domain containing the hairpins is also required for the oligomerization of these proteins, which may generate arc-like scaffolds to further stabilize the ER tubules [16]. In addition to these 2 protein families, proteins such as atlastins and their ortholog Sey1p in yeast may be involved in forming the tubular ER network [17].

Second, ER sheets assemble through the actions of proteins restricted to ER sheets, such as CLIMP-63, p180, and kinectin. CLIMP-63 is involved in attaching ER membranes and microtubules [18]. Knocking down CLIMP-63 reduces the luminal width of ER sheets, indicating that this protein maintains normal luminal width [19]. p180, which was first identified as a ribosome receptor [20], is also involved in the interaction between ER and microtubules [21], and p180 is further required for the expansion of the trans-Golgi network [22]. p180 is essential for anchoring ribosomes to the ER [23], and membrane-bound ribosomes are involved in gathering ER sheets and localizing certain membrane proteins to the ER sheets [19]. Kinectin, a binding partner of the microtubule motor protein kinesin [24], is required for assembling the translation elongation factor-1 complex on the ER [25]. Kinectin also regulates ER dynamics and contributes to ER shape formation [26], [27]. Interestingly, reticulons are localized at the edges of sheets where they stabilize the high curvature [19]. Thus, ER sheet formation is likely determined by a tug-of-war between sheet-promoting proteins and curvature-stabilizing proteins. Intriguingly, most of the key proteins of ER sheets contain coiled-coil and transmembrane domains that mediate protein-protein interactions and ER localization, respectively [21], [28]–[30]. Thus, these domains may represent a general feature of ER proteins.

Genes encoding putative proteins of the transmembrane and coiled-coil domain (TMCC) family have been found in many organisms. However, the properties and functions of TMCC proteins are unknown. TMCC1, a representative member of the TMCC family, also remains uncharacterized. However, the *tmcc1* locus in humans has been reported to be involved in hereditary congenital facial palsy [31], [32], although *tmcc1* may not be the causative gene [31], and TMCC1 mRNA and peptides have been identified in screening assays [33]–[36]. Here, we report that TMCC1 is an evolutionarily conserved protein. Using an antibody we raised against TMCC1, we first identified TMCC1 expression in diverse human cells. We also found that TMCC1 localized to the rough ER through its C-terminal transmembrane domains and associated with ribosomal proteins through its cytosolic region. Furthermore, TMCC1 was able to dimerize or oligomerize with TMCC proteins by using the large coiled-coil domain adjacent to the C-terminus. Our results suggest that TMCC1 functions in ER organization.

## Results

### Detection of TMCC1

**Figure 1:**
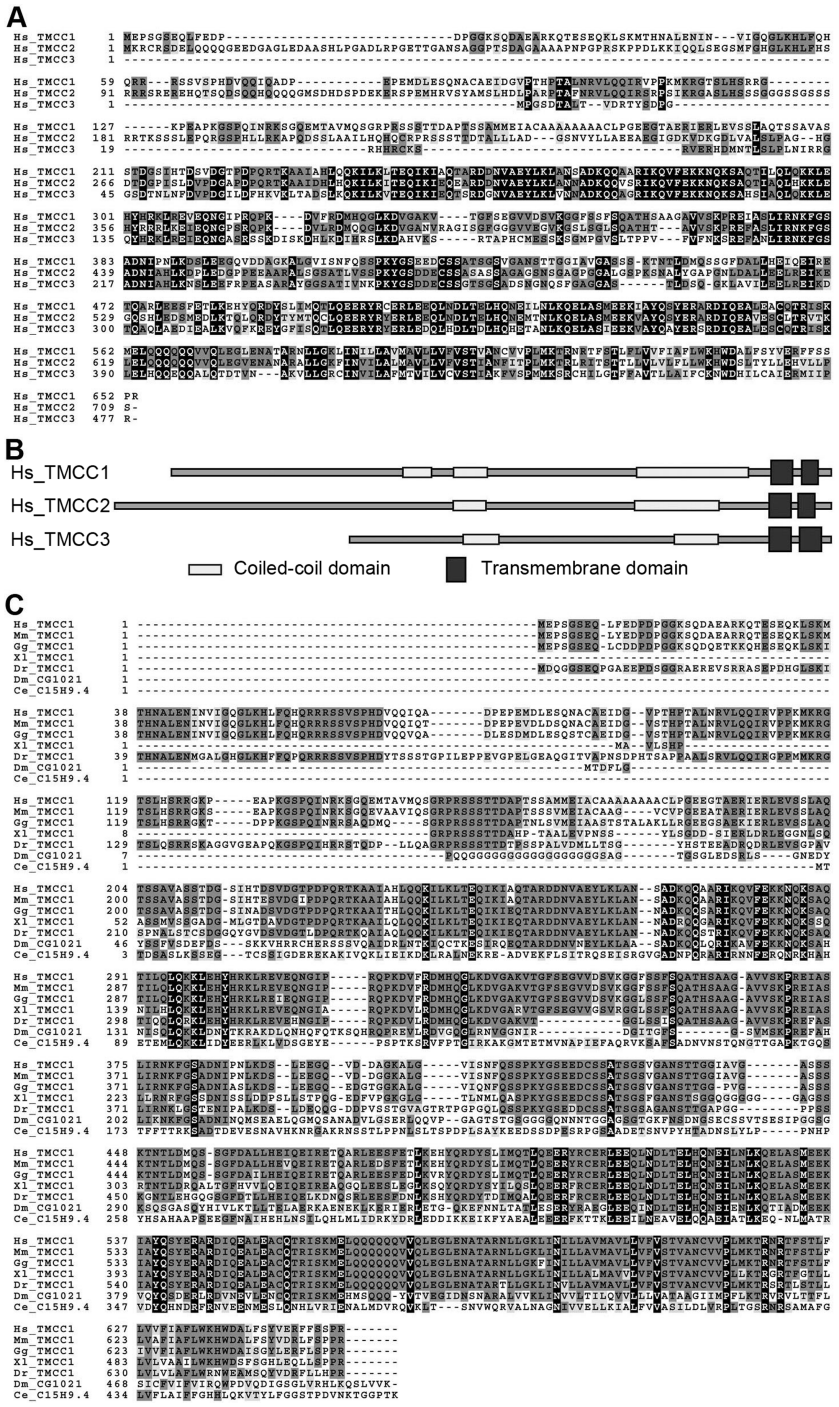
Sequence alignment of TMCC proteins. (A) Human TMCC family members. (B) Domain structures of human TMCC proteins. (C) TMCC1 in various organisms. Hs, *Homo sapiens*; Mm, *Mus musculus*; Gg, *Gallus gallus*; Xl, *Xenopus laevis*; Dr, *Danio rerio*; Dm, *Drosophila melanogaster*; Ce, *Caenorhabditis elegans*.

TMCC1 belongs to the TMCC family that includes at least 3 proteins in humans (TMCC1, 2, and 3). Sequence alignment showed that the 3 TMCCs have highly similar protein sequences (Fig. 1A), and that all contained the predicted coiled-coil and transmembrane domains (Fig. 1B). TMCC1 sequences are also found in other vertebrates such as mouse, chicken, frog, and zebrafish, and in lower organisms such as the fruit fly and the nematode *Caenorhabditis elegans*. Sequence alignment of TMCC1 from various organisms showed several conserved regions that share high sequence similarity (Fig. 1C). This evolutionary conservation of TMCC1 suggests that the protein is functionally important in most organisms.

**Figure 2:**
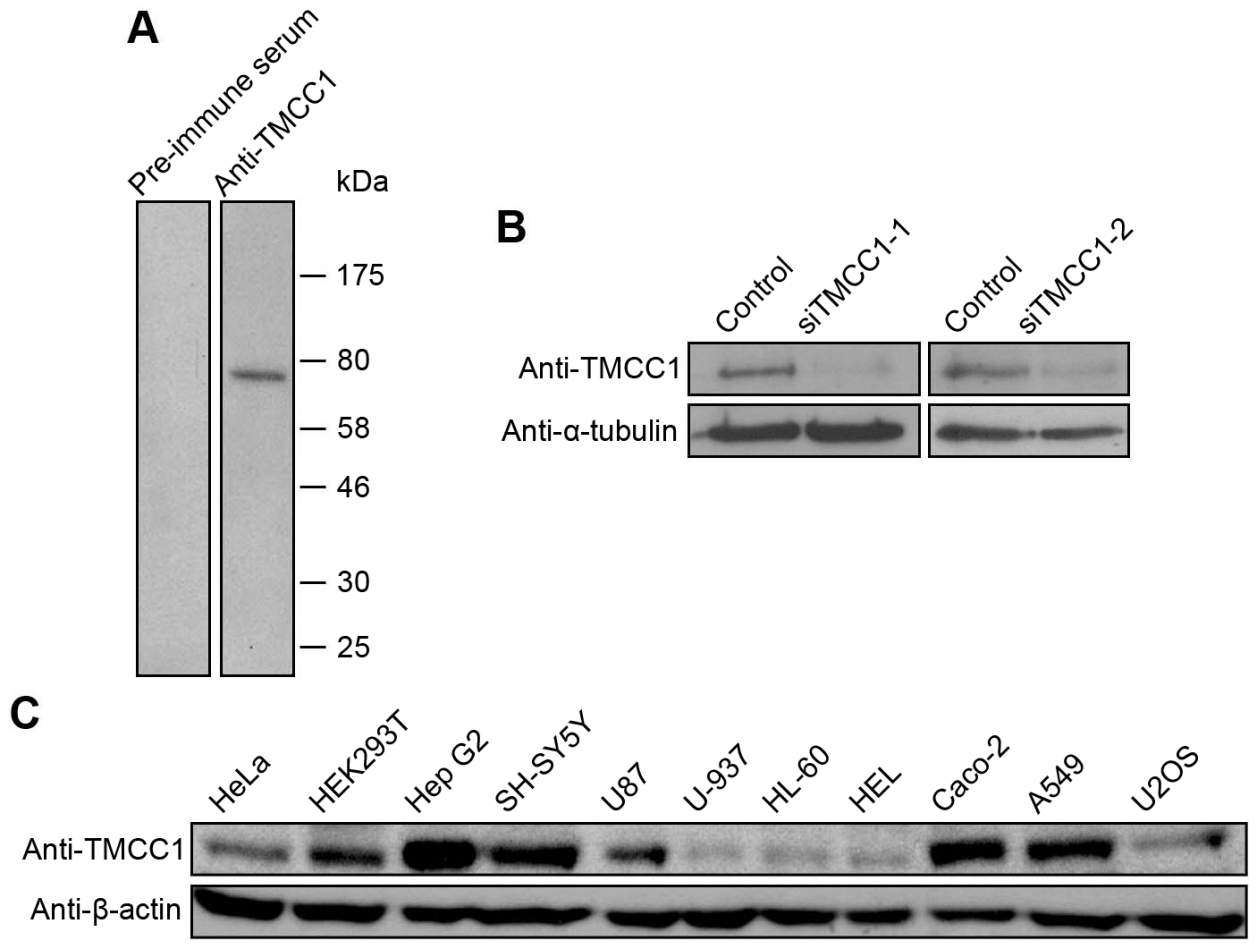
Expression of TMCC1 protein in human cells. (A) Whole cell extracts of HeLa cells were collected and immunoblotted with pre-immune serum or TMCC1 antibody. (B) HeLa cells were transfected with TMCC1 siRNA (siTMCC1-1 or siTMCC1-2) or a control siRNA; 72 h post-transfection, whole cell extracts were prepared and immunoblotted for TMCC1. α-Tubulin was stained as a control. (C) Whole cell extracts of various human cell lines were collected and immunoblotted for TMCC1; β-actin was stained as a control.

To identify the TMCC1 protein, we generated an anti-TMCC1 antibody in rabbits. An N-terminal fragment, TMCC1(1–200), was chosen as the antigen because this region is unique among TMCC family members in human; the protein fragment was also used to purify the polyclonal antibody against this region of TMCC1. In western-blotting experiments performed on whole cell extracts of HeLa cells, anti-TMCC1 recognized a protein band with a molecular weight similar to the theoretical molecular weight of TMCC1, and this band was not detected by the pre-immune serum (Fig. 2A). Moreover, in extracts of HeLa cells transfected with TMCC1 siRNAs, the level of the protein stained by anti-TMCC1 was decreased by over 80% (Fig. 2B). These results demonstrate that the TMCC1 antibody recognized endogenous TMCC1 specifically.

Next, we tested for TMCC1 protein expression in several human cell lines. As shown in Fig. 2C, TMCC1 protein was detected in all cell lines examined, with epithelial cells (Hep G2, Caco-2, and A549), neuroblastoma cells (SH-SY5Y), and glioblastoma cells (U87) showing high expression levels of TMCC1, and leukemia cells (HL-60 and HEL) and lymphoma cells (U-937) showing low expression levels. These results suggest that TMCC1 is expressed in diverse types of human cells.

### Subcellular localization of TMCC1

**Figure 3:**
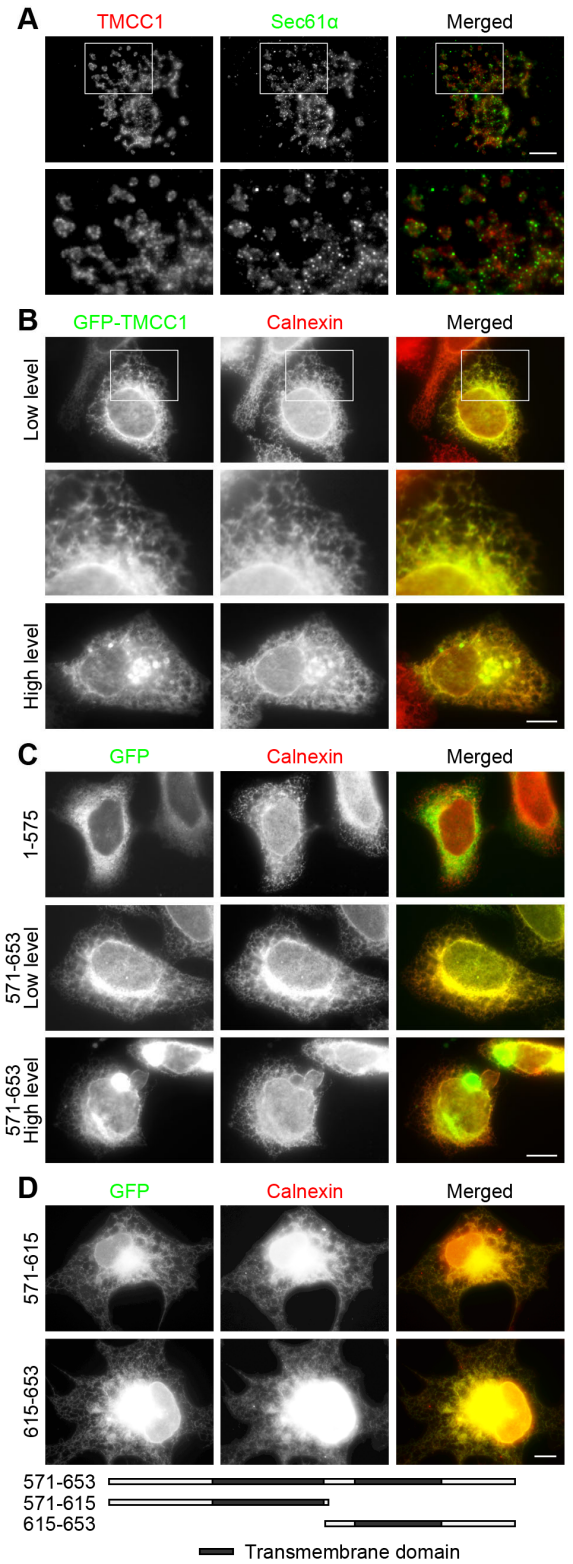
Subcellular localization of TMCC1. (A) Saponin-extracted COS-7 cells were fixed with methanol and stained with both Sec61α and TMCC1 antibodies; the boxed area shown is magnified. (B–C) HeLa cells were transfected with plasmids encoding GFP-tagged TMCC1 full-length protein, TMCC1(1–575), or TMCC1(571–653); 24 h post-transfection, cells with low and high levels of the exogenous proteins were fixed with methanol and stained with an anti-calnexin antibody. A magnified view of the boxed area in (B) is shown. (D) COS-7 cells were transfected with plasmids encoding GFP-tagged TMCC1(571–615) or TMCC1(615–653); 24 h post-transfection, cells were fixed with paraformaldehyde and then permeabilized with 0.2% Triton X-100 for 10 min at room temperature. Cells were then stained with an anti-calnexin antibody. Scale bars, 10 µm.

After identifying TMCC1 as a protein expressed widely in human cells, we examined the subcellular localization of TMCC1. We chose COS-7 cells for immunolabeling experiments because these cells are large. Labeling by anti-TMCC1 (Fig. 3A) showed that TMCC1 was present in the cytoplasm and in the nucleus and, furthermore, that TMCC1 was colocalized with Sec61α, a rough ER marker. Sec61α, a subunit of the Sec61 complex, associates tightly with membrane-bound ribosomes [37]. For this experiment, COS-7 cells were extracted with saponin before fixing with methanol to enhance the specificity of labeling by the Sec61α antibody.

Our immunolabeling results indicated that TMCC1 localized to the rough ER. To confirm this, we monitored the localization of TMCC1 transiently transfected into cells: HeLa cells were transfected with a plasmid encoding GFP-TMCC1 and then examined by fluorescence microscopy. When expressed at low levels, GFP-TMCC1 localized throughout the ER, showing an almost identical distribution as calnexin, an integral ER protein that is used widely as an ER marker (Fig. 3B). The localizations of transfected and endogenous TMCC1 were similar but not identical, which may be because of the higher levels of the ectopically expressed protein; Sec61β, a rough ER protein, was also found to be distributed throughout the ER when overexpressed in cells [19]. Thus, our results indicate that TMCC1 is a rough ER protein. When GFP-TMCC1 was expressed at high levels, the ER structure was deformed and clusters of calnexin were observed in these cells (Fig. 3B, bottom panel), suggesting that the expression levels of TMCC1 influence ER structure.

Because TMCC1 contains 2 adjacent transmembrane domains at the C-terminus, we investigated whether these domains were responsible for targeting TMCC1 to the ER or if other regions of the protein were also necessary. We transfected HeLa cells with plasmids to express either GFP-TMCC1 lacking the transmembrane domains (aa 1–575) or only the C-terminal transmembrane domains of TMCC1 (aa 571–653), and then stained the cells with calnexin antibody. GFP-TMCC1(1–575) localized in the cytosol and showed no specific pattern of distribution, whereas GFP-TMCC1(571–653) colocalized with calnexin (Fig. 3C), much like full-length GFP-TMCC1. Therefore, TMCC1(571–653) was identified as the ER-targeting domain of the protein, and this region was required and sufficient for ER localization. Moreover, in cells that expressed high levels of the TMCC1 transmembrane domains, the ER structure was deformed (Fig. 3C, bottom panel), much as it was in cells that expressed high levels of full-length TMCC1 (Fig. 3B, bottom panel). To rule out any potential effect from GFP, we expressed the transmembrane domains with a FLAG-tag and obtained the same results (Fig. S1). To examine the potential role of each transmembrane domain in TMCC1, we transfected COS-7 cells with plasmids encoding single transmembrane domains of TMCC1 and checked their localization. As shown in Fig. 3D, both GFP-tagged TMCC1(571–615) and TMCC1(615–653) colocalized with calnexin, indicating that each of the transmembrane domains possessed the ER-targeting property.

**Figure 4:**
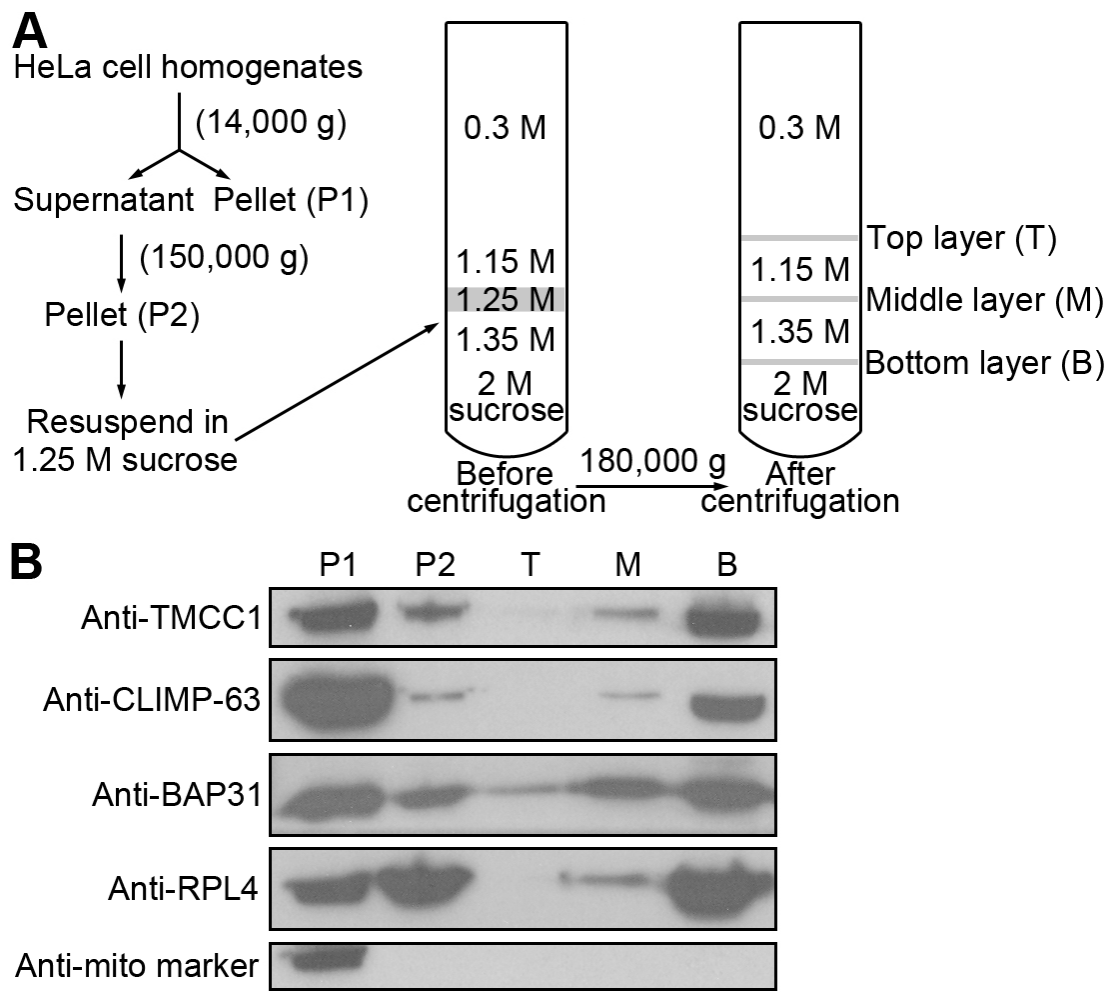
ER isolation. (A) Workflow of ER isolation. HeLa cells were homogenized in 0.3 M sucrose. After 2 centrifugations, the P2 pellet was resuspended in 1.25 M sucrose and subjected to discontinuous sucrose-gradient centrifugation, and then the distinct layers at interfaces were collected. (B) Various fractions from (A) were collected and immunoblotted for ER, ribosomal, and mitochondrial proteins.

To confirm the ER localization of TMCC1 further, we isolated ER proteins from cells: HeLa cells were homogenized and ER proteins were purified using discontinuous sucrose-gradient centrifugation (Fig. 4A). Because of the presence of membrane-bound ribosomes, rough ER has a high density and most of it accumulates in the bottom layer of gradients, whereas smooth ER accumulates in the top layer [38]. As shown in Fig. 4B, most of the rough ER protein CLIMP-63 and the ribosomal protein RPL4 were detected in the bottom layer of our sucrose gradients, but the integral ER protein BAP31 was detected in all the layers. By contrast, most of the mitochondrial protein was present only in the P1 fraction. TMCC1 showed the same distribution as CLIMP-63 and RPL4, but not BAP31, indicating that TMCC1 is a rough ER protein. Taken together, our findings showed that TMCC1 localized to the rough ER and that TMCC1 was targeted to the ER by the C-terminal transmembrane domains.

### Topology of TMCC1

**Figure 5:**
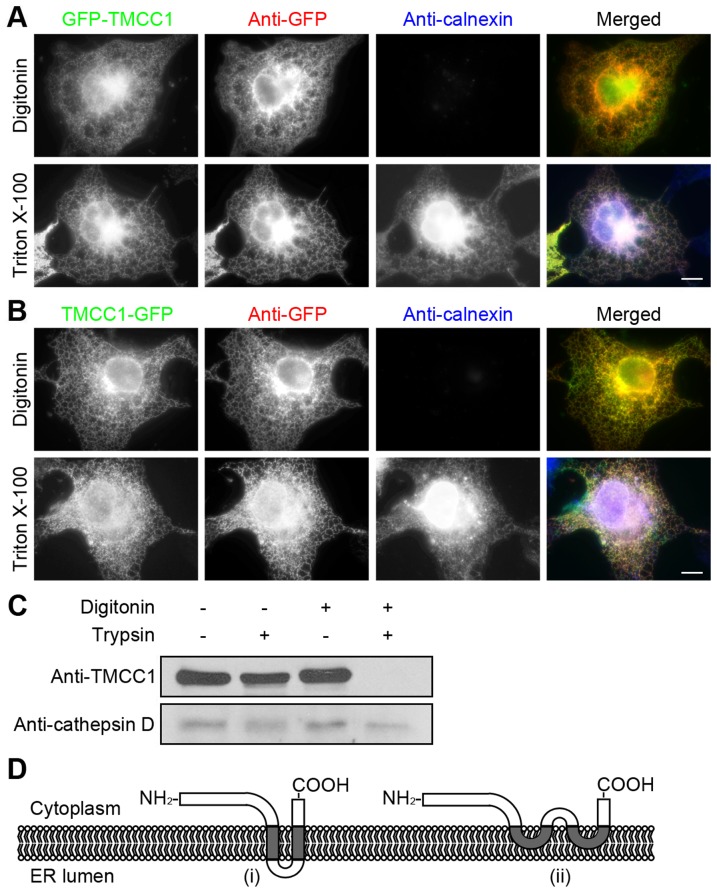
Topology of TMCC1. (A–B) COS-7 cells were transfected with a plasmid encoding N-terminal (A) or C-terminal (B) GFP-tagged TMCC1; 24 h post-transfection, cells were fixed with paraformaldehyde and then permeabilized with either 40 µg/mL digitonin for 5 min on ice or 0.2% Triton X-100 for 10 min at room temperature. Cells were then co-stained with GFP and calnexin antibodies. Scale bars, 10 µm. (C) HeLa cells were treated with various combinations of digitonin and trypsin and then immunoblotted for TMCC1 and cathepsin D. (D) Two possible models of TMCC1 topology. Model (i) shows a transmembrane topology with 2 transmembrane domains, and Model (ii) shows an intramembrane topology with 2 intramembrane domains.

After identifying the C-terminal transmembrane domains of TMCC1 as the potential ER-targeting region of the protein, we sought to examine the topology of the N-terminal region of TMCC1. We transfected TMCC1 with GFP tagged to the N-terminus into COS-7 cells and then immunolabeled these cells. The cells were first fixed with paraformaldehyde and then permeabilized with either digitonin or Triton X-100. Digitonin selectively permeabilizes the plasma membrane and leaves other membranes intact, whereas Triton X-100 permeabilizes cellular membranes non-selectively. The fixed and permeabilized cells were labeled with GFP and calnexin antibodies. Calnexin has a large ER luminal domain and a short cytoplasmic tail. Because the monoclonal calnexin antibody recognizes an epitope present within the ER lumen, and the ER membrane was intact in digitonin-permeabilized cells, calnexin was detected only in cells permeabilized with Triton X-100 (Fig. 5A). By contrast, the N-terminal GFP tag of the exogenous TMCC1 protein was detected both in digitonin- and Triton X-100-permeabilized cells (Fig. 5A), indicating that the N-terminal region of TMCC1 faces the cytoplasm and not the ER lumen. Moreover, we also examined the topology of the C-terminal tail of TMCC1 by using the same assay. COS-7 cells were transfected with plasmid encoding TMCC1 with C-terminal GFP tag. After paraformaldehyde fixation and detergent permeabilization, cells were labeled with GFP and calnexin antibodies. As shown in Fig. 5B, the C-terminal GFP tag was also detected both in digitonin- and Triton X-100-treated cells, indicating that the C-terminal tail of TMCC1 also resides in the cytoplasm.

To further confirm the above result, we performed protease protection assays. HeLa cells were incubated with digitonin to permeabilize the plasma membrane, and then treated with trypsin to digest exposed proteins. Under these conditions, the control protein cathepsin D, which is an aspartyl protease present within lysosomes, was not digested by trypsin because it was protected by the lysosomal membrane. By contrast, TMCC1 was digested by trypsin, indicating that the N-terminus of TMCC1 was present in the cytoplasm (Fig. 5C). Thus, the above results together indicated that both of the N-terminal region and the C-terminal tail of TMCC1 reside in the cytoplasm. Based on these results, 2 possible models of TMCC1 topology are presented in Fig. 5D.

### Interaction between TMCC proteins

**Figure 6:**
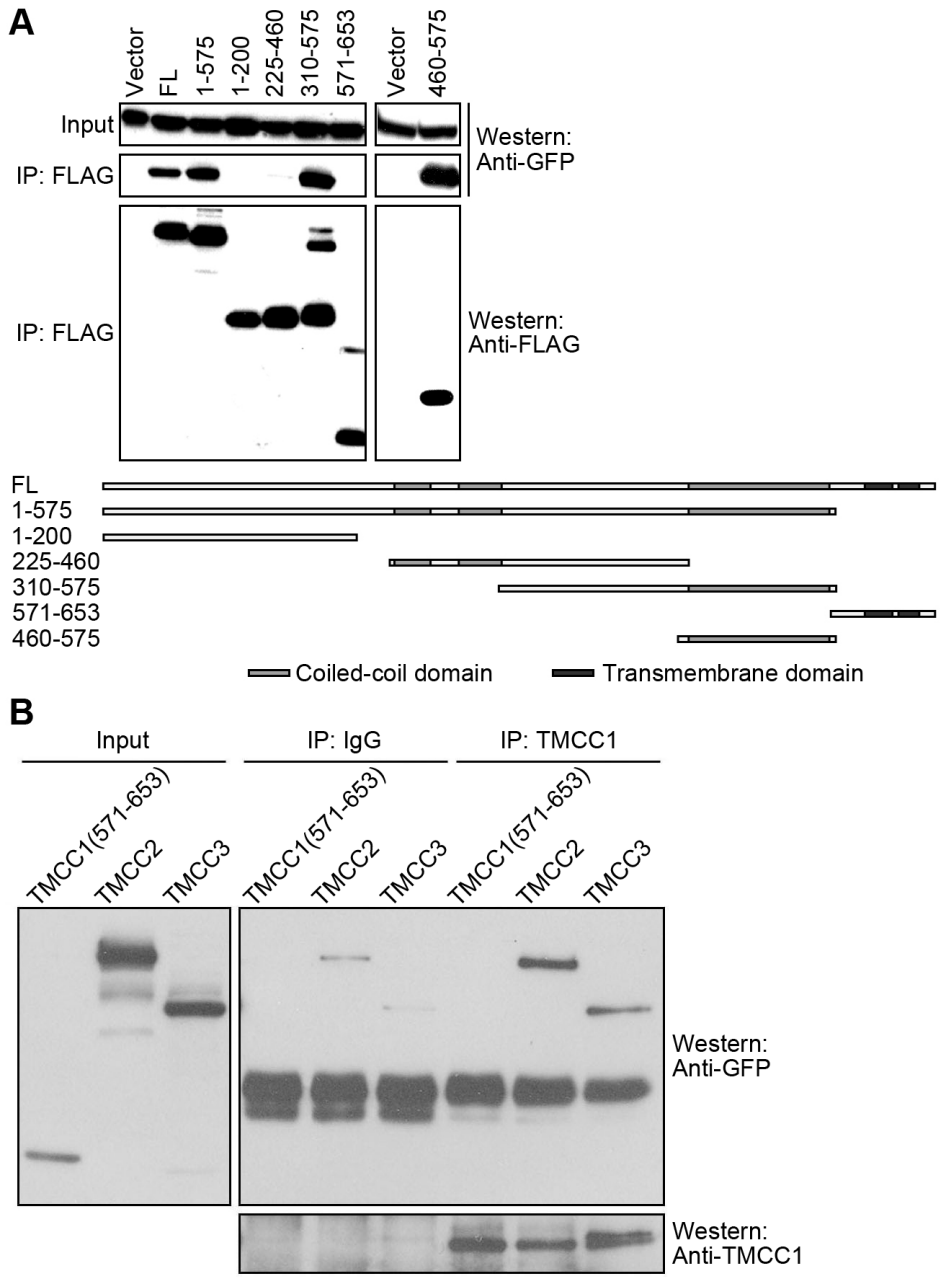
Homo- or hetero-dimerization or oligomerization of TMCC proteins. (A) HEK293T cells were co-transfected with plasmids encoding GFP-TMCC1 and FLAG-tagged TMCC1 fragments; 24 h post-transfection, cell lysates were collected for anti-FLAG immunoprecipitation to test for interactions between FLAG- and GFP-tagged proteins by performing western blotting. A schematic representation of the TMCC1 constructs is presented alongside the blots. Vector, pFLAG-CMV2 vector. FL, full-length TMCC1. (B) HEK293T cells were transfected with GFP-tagged TMCC1(571–653), TMCC2, or TMCC3 plasmids; 24 h post-transfection, cell lysates were prepared for TMCC1 immunoprecipitation to test for interaction between TMCC1 and exogenous proteins. TMCC1 and GFP-tagged proteins were analyzed by western blotting.

Coiled-coil domains are known to mediate protein-protein interactions, and several ER proteins containing coiled-coil domains are thought to form oligomers by using these domains [14]-[16], [19]. To determine whether the coiled-coil domains of TMCC1 function similarly, we conducted immunoprecipitation experiments. HEK293T cells were transfected with plasmids containing sequences of full-length FLAG-TMCC1 and GFP-tagged TMCC1, and lysates prepared from these cells were used for immunoprecipitation with anti-FLAG antibody. Western blotting showed that GFP-TMCC1 was pulled down by FLAG-tagged full-length TMCC1 (Fig. 6A), which indicates intermolecular interaction between the TMCC1 proteins. Furthermore, we tested the interaction between GFP-TMCC1 and a range of FLAG-tagged TMCC1 fragments (Fig. 6A). Only the fragments containing the large coiled-coil domain, TMCC1 460–575, 310–575, and 1–575, pulled down GFP-TMCC1 (Fig. 6A). Therefore, TMCC1 was able to dimerize or oligomerize and this interaction required its large coiled-coil domain adjacent to the C-terminus.

Because the coiled-coil domain adjacent to the C-terminus of TMCC1 is highly conserved among TMCC family members and this domain is required for intermolecular interaction between TMCC1 proteins, we tested whether TMCC1 interacts with other TMCC proteins. We transfected HEK293T cells with plasmids encoding GFP-tagged TMCC2 or TMCC3 and then immunoprecipitated endogenous TMCC1. Rabbit IgG was used as the negative control for immunoprecipitation, and GFP-TMCC1(571–653) served as the negative control for the interaction of exogenous proteins with endogenous TMCC1 because this fragment did not interact with full-length TMCC1 (Fig. 6A). Both GFP-TMCC2 and GFP-TMCC3 co-immunoprecipitated with endogenous TMCC1, whereas GFP-TMCC1(571–653) did not (Fig. 6B), indicating that TMCC1 can also dimerize or oligomerize with other members of the TMCC family. Thus, using this coiled-coil domain, TMCC1 may form homo- or hetero-dimers or oligomers with other TMCC proteins.

### Interaction of TMCC1 with ribosomal proteins

**Figure 7:**
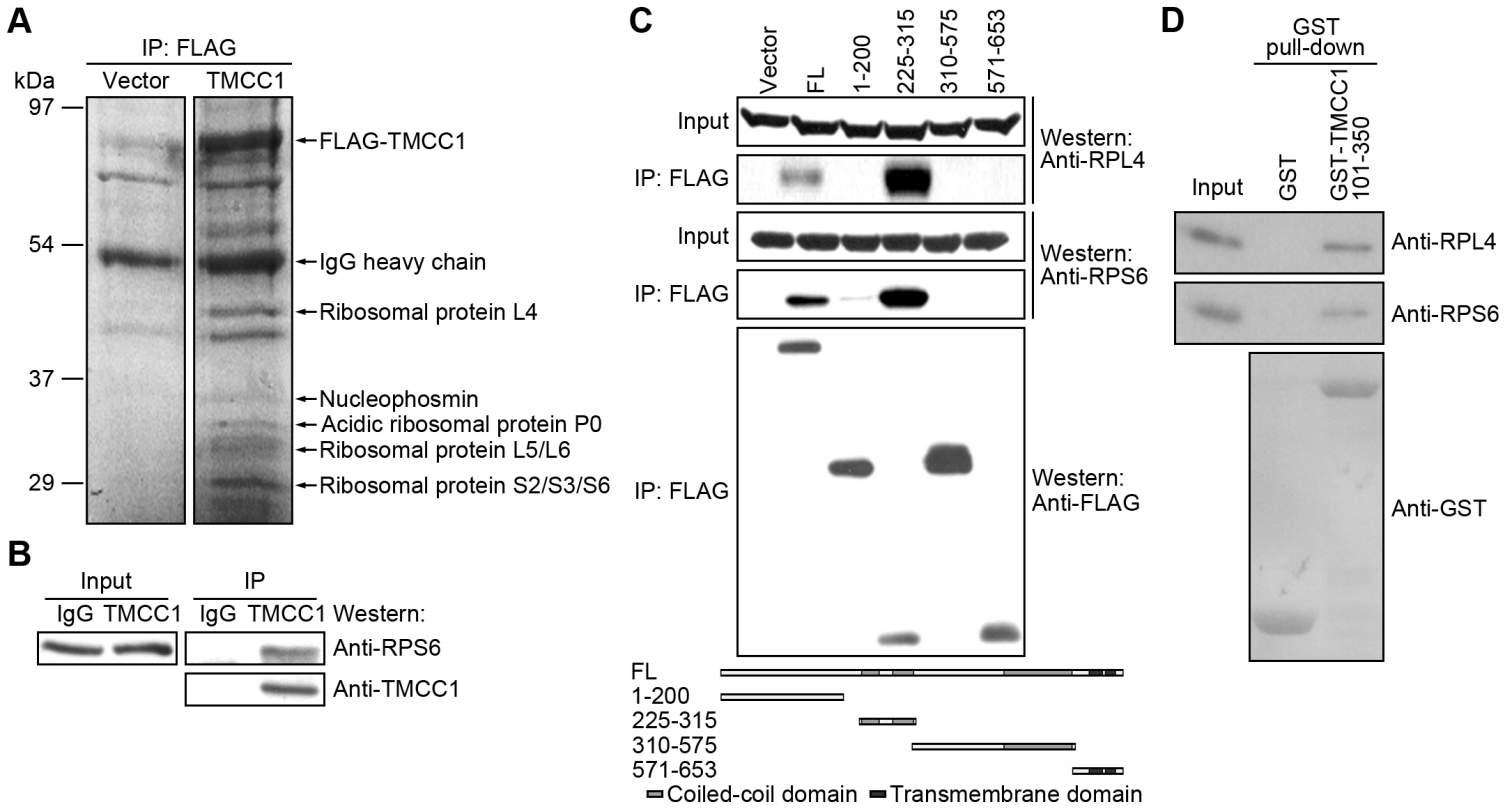
Interaction of TMCC1 with ribosomal proteins. (A) HEK293T cells were transfected with FLAG-tagged TMCC1 plasmid or the vector; 24 h post-transfection, cell lysates were prepared for anti-FLAG immunoprecipitation. Immunoprecipitated proteins were visualized on the protein gel by staining with Coomassie Brilliant Blue R-250. The protein bands marked in the figure were identified by mass spectrometry. Vector, pFLAG-CMV2 vector. (B) HeLa cell lysates were collected for TMCC1 immunoprecipitation, and samples were immunoblotted for TMCC1 and the ribosomal protein RPS6. (C) HEK293T cells were transfected with plasmids encoding FLAG-tagged TMCC1 full-length protein or fragments; 24 h post-transfection, cell lysates were collected for anti-FLAG immunoprecipitation. Ribosomal and FLAG-tagged proteins were analyzed by western blotting. Vector, pFLAG-CMV2 vector. FL, full-length TMCC1. (D) Ribosomes prepared from HeLa cells were incubated with purified GST or GST-TMCC1(101–350) protein and then pulled down using GSH-beads; ribosomal and GST-tagged proteins were analyzed by western blotting.

To understand the functions of TMCC1, we performed mass spectrometry to identify TMCC1-binding proteins. HEK293T cells were transfected with the FLAG-TMCC1 plasmid, and cell lysates were used for anti-FLAG immunoprecipitation. Immunoprecipitated proteins were resolved by SDS-PAGE and stained with Coomassie Brilliant Blue R-250, and protein bands were identified using mass spectrometry. As shown in Fig. 7A, various ribosomal proteins, as well as nucleophosmin, were pulled down by FLAG-TMCC1. To confirm the interactions of these proteins, we immunoprecipitated endogenous TMCC1 from HeLa cell lysates: the ribosomal protein RPS6 co-immunoprecipitated with TMCC1 (Fig. 7B), indicating that TMCC1 interacts with ribosomal proteins. To identify the ribosome-binding domain of TMCC1, we transfected HEK293T cells with plasmids encoding various FLAG-TMCC1 fragments and repeated the anti-FLAG immunoprecipitation. Ribosomal proteins RPL4 and RPS6 were pulled down only by FLAG-TMCC1(225–315) and full-length TMCC1 (Fig. 7C). TMCC1(225–315) contains 2 adjacent short coiled-coil domains, and thus we conclude that these coiled-coil domains are responsible for the interaction with ribosomal proteins. To evaluate whether the interaction between TMCC1 and ribosomal proteins is direct or not, we performed ribosome-binding assays by using ribosomes purified from HeLa cells and GST-TMCC1(101–350) from *Escherichia coli*. As shown in Fig. 7D, GST-TMCC1(101–350) pulled down RPL4 and RPS6, whereas GST protein alone did not, suggesting that TMCC1 directly interacts with ribosomal proteins. Because TMCC1 is an ER membrane protein, these results also suggest that TMCC1 facilitates the attachment of ribosomes to the ER membrane.

## Discussion

We have shown that TMCC1 is an evolutionarily conserved protein and have provided first evidence of TMCC1 expression in human cells. Using immunolabeling and ER-isolation experiments, we have demonstrated that TMCC1 is a rough ER protein. The C-terminal transmembrane domains of TMCC1 were shown to target the protein to the ER, and the N-terminal region and C-terminal tail of TMCC1 were shown to face the cytoplasm. Furthermore, we have demonstrated that TMCC1 can interact with TMCC proteins and with ribosomal proteins, and that TMCC1 overexpression deforms the ER. Therefore, we conclude that TMCC1 is a rough ER protein that may regulate ER membrane organization and the attachment of ribosomes to the ER.

TMCC1 localization in rough ER was demonstrated by immunolabeling and also by isolating ER proteins. We observed almost identical labeling patterns for GFP-TMCC1 and calnexin by immunostaining. In the magnified immunofluorescence micrographs, the signals of GFP-TMCC1 overlapped with, but were not exactly merged with, those of calnexin (Fig. 3B). The difference in TMCC1 and calnexin patterns may be a result of their distinct topologies: TMCC1 possesses a large cytosolic N-terminal region and GFP was tagged to the N-terminus, whereas calnexin has a large ER luminal domain and a short cytoplasmic tail. Therefore, the signals of GFP-TMCC1 originated from the cytosolic face of the ER and calnexin was stained on the luminal face of the ER. By comparing the distribution patterns of distinct TMCC1 fragments, we confirmed that the C-terminal transmembrane domains were responsible for ER targeting. In line with the general properties of transmembrane domains, the ER-targeting domain of TMCC1 may be inserted into the ER membrane and TMCC1 might reside as an integral membrane protein on the ER. However, the transmembrane domains of TMCC1 appear to define a novel ER-targeting motif, because these domains do not show sequence similarity to known ER proteins. Because the transmembrane domains are highly conserved in TMCC family members, the other TMCC proteins are also likely to be ER proteins. This notion is consistent with a recent study showing TMCC2 localization [39] and it is supported by our observations on the distribution of transiently transfected TMCC2 and TMCC3 (Fig. S2).

By immunostaining, we found that each transmembrane domain of TMCC1 localized to the ER (Fig. 3D). We BLAST-searched sequence databases with the transmembrane domain sequences of TMCC1, and found that the second transmembrane domain shows homology to the transmembrane domains of the ER proteins atlastin-1 and translocon-associated protein subunit γ. Between the 2 transmembrane domains of TMCC1, a short 6-amino-acid segment is present. Because of its hydrophilicity, this segment is not likely to be embedded within the ER membrane, but we have not obtained any evidence thus far indicating the topology of this segment. Two possible models of TMCC1 topology with the N-terminal region and C-terminal tail residing in the cytoplasm are shown in Fig. 5D. Model (i) shows a 2-pass Type III transmembrane topology, as per the nomenclature [40]. In this model, the first transmembrane domain functions as the signal-anchoring sequence, and the second functions as the stop-transfer anchor sequence. Model (ii) shows an intramembrane topology rather than a transmembrane topology. Further studies are required to clarify the overall topology of TMCC1.

When TMCC1 transmembrane domains and full-length protein were transiently transfected into cells and expressed at high levels, the ER structure was deformed. Overexpression of several ER-membrane proteins has been reported to cause similar defects in ER morphology [41]–[43]. Moreover, even the overexpression of a nuclear envelope protein can affect ER structure [44]. In these cases, the cytoplasmic regions of the proteins appeared to be required for causing the ER defect; with TMCC1, however, we found that the transmembrane domains alone could induce ER deformation. Therefore, TMCC1 may affect ER structure through a mechanism that differs from the mechanism(s) used by other proteins of the ER membrane [41]–[43] and nuclear envelope [44]. The formation of the normal ER structure requires proper membrane curvature. The overexpressed TMCC1 transmembrane domains may affect the curvature of the ER membrane directly, or the TMCC1 accumulated in the ER membrane may affect the distribution of other curvature-stabilizing proteins to alter membrane curvature and deform the ER.

Our selective-permeabilization experiments using digitonin showed that the N-terminal region of TMCC1 resides in the cytoplasm and not in the ER lumen. Thus, the long, cytoplasmic N-terminal region of TMCC1 may bind to diverse targets much like other ER proteins [21], [23], [30], and TMCC1 may recruit its binding partners to the ER membrane. In the cytoplasmic region, the small tandem coiled-coil domains interact with ribosomal proteins such as RPL4 and RPS6, suggesting that TMCC1 helps attach ribosomes to the ER membrane. RPL4 is a component of the 60S subunit of ribosomes, and in *E. coli*, this protein stimulates transcription termination in the S10 operon leader [45]. RPS6 is a component of the 40S subunit of ribosomes, and the phosphorylation of RPS6 may be involved in the regulation of protein synthesis, cell size, and glucose homeostasis [46]. Nucleophosmin, an abundant nucleolar phosphoprotein [47], was identified by mass spectrometry as a TMCC1-binding protein. Nucleophosmin interacts directly with several ribosomal proteins [48]–[50] and is critical for the nuclear export of ribosomal proteins [50], suggesting that TMCC1 may also be involved in ribosomal biogenesis. Moreover, the coiled-coil domain adjacent to the transmembrane domains in the cytoplasmic region interacts with TMCC proteins to form homo- and hetero-dimers or oligomers. Because the coiled-coil domain is highly conserved among TMCC proteins, this domain in TMCC2 and TMCC3 may also mediate the dimerization or oligomerization. These TMCC dimers or oligomers could potentially be poorly mobile and similar to CLIMP-63 [29], and thus might regulate membrane motility or protein mobility locally. If TMCC1 interacts with TMCC proteins from apposing membranes, the proteins might help establish inter-membrane connections and communication. Moreover, oligomerization could also regulate the interaction between TMCC1 and its binding partners.

In human, TMCC family includes at least 3 members. As shown in Fig. 1, the TMCC members contain a variable region (e.g. ∼200 aa in TMCC1) at the N-terminus and the rest of the proteins is highly homologous among the members. The variable region may bestow distinct properties in the TMCCs. We analyzed the TMCC sequences but did not identify any recognized motif or domain within the variable region. Therefore, the function of the variable region remains unknown.

In summary, we have characterized TMCC1, a member of the conserved TMCC family, and have shown that TMCC1 is an integral ER-membrane protein. Consistent with these results, the overexpression of TMCC1 or its transmembrane domains perturbed ER organization. However, we did not observe any substantial defect in ER morphology after RNAi-mediated suppression of TMCC1 expression, which may be because of the presence of other TMCC members. We have also identified the association of TMCC1 with ribosomal proteins. Thus, TMCC proteins may help recruit proteins such as those associated with ribosomes to the ER membrane and thereby regulate ER organization.

## Materials and Methods

### Plasmids

The cDNA clones of human TMCC1 (Accession No.: NM_001017395) and TMCC3 (Accession No.: NM_020698) were purchased from American Type Culture Collection (I.M.A.G.E. Clone ID: 5527623 and 5264859). The cDNA clone of human TMCC2 (Accession No.: NM_014858) was obtained from Kazusa DNA Research Institute, Japan. The coding sequences of the TMCCs were cloned into pEGFP-C1 (Clontech), pEGFP-N3 (Clontech), or pFLAG-CMV2 (Sigma-Aldrich). TMCC1 fragment aa 1–200 was cloned into pET-28a(+) (Novagen). TMCC1 fragments aa 1–200 and 101–350 were cloned into pGEX-4T-3 (GE Healthcare). TMCC1 fragments aa 1–575, 571–653, 571–615, and 615–653 were cloned into pEGFP-C1. TMCC1 fragments aa 1–575, 1–200, 225–460, 310–575, 571–653, 460–575, and 225–315 were cloned into pFLAG-CMV2.

### Antibodies

To generate antibodies against TMCC1, a fragment of TMCC1, 1–200, was expressed in *E. coli* BL21 (DE3) as a fusion with either a 6xHis or a glutathione S-transferase (GST) tag. The 6xHis and GST fusion proteins were purified using Ni^2+^-nitrilotriacetic acid resin (Qiagen) and GSH-beads (GE Healthcare), respectively. The 6xHis-tagged TMCC1 fragment was used to immunize rabbits, and the antibody produced against TMCC1 was purified from rabbit sera using the GST-tagged protein immobilized on nitrocellulose membranes (Pall). Mouse monoclonal antibodies against FLAG (M2), α-tubulin (DM1A), β-actin (AC-15), and a rabbit polyclonal antibody against FLAG were obtained from Sigma-Aldrich. Normal rabbit IgG, a rabbit polyclonal antibody against GFP (FL), and a goat polyclonal antibody against Sec61α (G-20) were from Santa Cruz Biotechnology. A mouse monoclonal antibody against mitochondria (MTC02) was purchased from Abcam, and a goat polyclonal antibody against GST was from Amersham Pharmacia Biotech. A mouse monoclonal antibody against CLIMP-63 (G1/296) was obtained from Alexis, and a mouse monoclonal antibody against RPL4 (4A3) was from Abnova. Mouse monoclonal antibodies against calnexin and BAP31 were kindly provided by Cancer Institute and Hospital, Tianjin, China. A rabbit polyclonal antibody against cathepsin D (Ab-2) was from Calbiochem and a rabbit polyclonal antibody against RPS6 was from Cell Signaling Technology. Secondary antibodies conjugated with Alexa Fluor dyes (Alexa Fluor 488, 594, or 647) were purchased from Invitrogen.

### Cell culture, transfection, and RNA interference

HEK293T, HeLa, U2OS, and COS-7 cells were cultured in Dulbecco’s Modified Eagle Medium supplemented with 10% fetal bovine serum (FBS). Hep G2, U-937, and HEL cells were cultured in RPMI 1640 Medium supplemented with 10% FBS. SH-SY5Y and U87 cells were cultured in Eagle’s Minimum Essential Medium (EMEM) supplemented with 10% FBS. Caco-2 cells were cultured in EMEM supplemented with 20% FBS. HL-60 cells were cultured in Iscove’s Modified Dulbecco’s Medium supplemented with 20% FBS. A549 cells were cultured in F-12K Medium supplemented with 10% FBS. All cells were obtained from the American Type Culture Collection and grown at 37°C in a humidified atmosphere with 5% CO_2_. Plasmids were transfected into HEK293T cells by using Lipofectamine and Plus reagents (Invitrogen). Lipofectamine 2000 (Invitrogen) was used for transfecting plasmids and small interfering RNAs (siRNAs) into HeLa and COS-7 cells. Two siRNA duplexes targeting TMCC1 were synthesized by Shanghai GenePharma: siTMCC1-1, CGAUUGGAAGAACAGCUAA; siTMCC1-2, GCAGACAGAAUCAGAACAA.

### Immunofluorescence microscopy

Cells grown on glass coverslips were fixed with cold methanol at -20°C for 5 min or with 4% paraformaldehyde in phosphate-buffered saline (PBS) at room temperature for 15 min. After fixation, cells were labeled with primary antibodies and subsequently with Alexa Fluor dye-conjugated secondary antibodies. Nuclei were labeled with Hoechst 33258 (Sigma-Aldrich). Coverslips were mounted with Mowiol (Calbiochem) and examined using an inverted fluorescence microscope (Eclipse TE2000-E; Nikon). To label Sec61α, COS-7 cells were extracted with 1 mg/mL saponin in PBS for 5 min at room temperature before fixing with methanol.

### ER isolation

The protocol for isolating ER was adapted from methods described elsewhere [38]. HeLa cells were homogenized in ice-cold homogenization buffer (10 mM HEPES-KOH, pH 7.0, 0.3 M sucrose, 2 mM dithiothreitol, and protease inhibitor cocktail) by using a Kontes 7-mL glass homogenizer. The homogenates were centrifuged at 14,000 × *g* for 20 min at 4°C to remove cell debris, nuclei, and mitochondria. The supernatant was then centrifuged at 150,000 × *g* for 30 min at 4°C in an ultracentrifuge (Beckman Coulter) using a TLA-100.4 rotor to obtain a total microsomal pellet. The pellet was resuspended in ice-cold resuspension buffer (10 mM HEPES-KOH, pH 7.0, 1.25 M sucrose, 2 mM dithiothreitol, and protease inhibitor cocktail) by using a Kontes 18 glass homogenizer. Next, a discontinuous sucrose gradient was prepared in centrifuge tubes with the following solutions layered from top to bottom: 1.2 mL of 0.3 M sucrose, 0.3 mL of 1.15 M sucrose, 0.1 mL of 1.25 M sucrose containing the total microsomes, 0.3 mL of 1.35 M sucrose, and 0.4 mL of 2 M sucrose. The tubes were centrifuged at 180,000 × *g* for 90 min at 4°C using a TLS-55 swinging-bucket rotor. The material at each interface was diluted to 0.3 M sucrose and collected by centrifugation at 150,000 × *g* for 30 min at 4°C. These samples were analyzed by western blotting.

### Protease protection assay

HeLa cells were incubated with or without 20 µM digitonin (Sigma-Aldrich) in KHM buffer (20 mM HEPES-KOH, pH 7.3, 110 mM potassium acetate, and 2 mM MgCl_2_) for 5 min at room temperature, and then treated in the absence or presence of 0.25% trypsin for 5 min at 37°C. Reactions were stopped by adding a protease inhibitor cocktail (Roche Applied Science), and samples were analyzed by western blotting.

### Immunoprecipitation

HEK293T cells were lysed on ice using a lysis buffer (10 mM Tris-HCl, pH 7.4, 150 mM NaCl, 1 mM MgCl_2_, 1 mM EDTA, 1% Triton X-100, and protease inhibitor cocktail) and clarified by centrifugation. To immunoprecipitate TMCC1, Protein A Agarose beads (Invitrogen) were pre-incubated with normal rabbit IgG or the TMCC1 antibody at 4°C, and then incubated with cell lysates. To immunoprecipitate FLAG-tagged proteins, anti-FLAG M2 Agarose beads (Sigma-Aldrich) were added directly to cell lysates and incubated at 4°C. After incubation, the beads were washed with lysis buffer and boiled in electrophoresis sample buffer. Samples were resolved by SDS-PAGE and analyzed by western blotting.

### Mass spectrometry

Protein gels were stained with Coomassie Brilliant Blue R-250 (Sigma-Aldrich), and protein bands were excised from the gels, reduced, alkylated, and digested in-gel with trypsin [51]. Recovered peptides were analyzed by mass spectrometry as described previously [52].

### Ribosome-binding assay

GST and GST-TMCC1(101–350) proteins were purified using GSH-beads. The protocol for ribosome preparation was adapted from methods kindly provided by Dr. Alan M. Lin (National Yang-Ming University). HeLa cells were homogenized in ice-cold buffer A (20 mM Tris-HCl, pH 7.5, 50 mM KCl, 12.5 mM MgCl_2_, 0.25 M sucrose, and protease inhibitor cocktail) by using a Kontes 7-mL glass homogenizer. The homogenates were centrifuged at 3,000 × *g* for 30 min and then at 14,000 × *g* for 30 min at 4°C to remove cell debris, nuclei, and mitochondria. The supernatant was then centrifuged at 270,000 × *g* for 1 h at 4°C in an ultracentrifuge using a TLA-100.4 rotor. The pellet was resuspended in ice-cold buffer B (20 mM Tris-HCl, pH 7.5, 50 mM KCl, 12.5 mM MgCl_2_, 1% Triton X-100, 0.5% sodium deoxycholate, and protease inhibitor cocktail) by vigorously vortexing for 30 min at 4°C, and then the sample was centrifuged at 14,000 × *g* for 15 min at 4°C. The supernatant was carefully laid on the top of buffer C (20 mM Tris-HCl, pH 7.5, 50 mM KCl, 12.5 mM MgCl_2_, and 1 M sucrose) in a centrifuge tube, and then was centrifuged at 270,000 × *g* for 1 h at 4°C to collect ribosomes. The ribosomes were resuspended in ice-cold buffer D (20 mM Tris-HCl, pH 7.5, 100 mM NaCl, 12.5 mM MgCl_2_, 1 mM EDTA, 1 mM dithiothreitol, 0.2% Triton X-100, 1 mg/mL bovine serum albumin, and protease inhibitor cocktail), incubated with GST or GST-TMCC1(101–350) at 4°C, and then pulled down using GSH-beads. The pull-down samples were analyzed by western blotting.

## Supporting Information

Figure S1 **ER defects induced by overexpression of TMCC1 transmembrane domains.** COS-7 cells were transfected with a plasmid encoding FLAG-tagged TMCC1(571–653); 24 h post-transfection, cells with low and high levels of exogenous proteins were fixed with methanol and co-stained with FLAG and calnexin antibodies. Scale bar, 10 µm.
(TIF)

Figure S2 **Subcellular localization of TMCC2 and TMCC3.** COS-7 cells were transfected with plasmids encoding GFP-tagged TMCC2 or TMCC3; 24 h post-transfection, cells were fixed with methanol and stained with an anti-calnexin antibody. Scale bar, 10 µm.
(TIF)
