# Supplementary figures and images for: Transmembrane and Coiled-Coil Domain Family 1 Is a Novel Protein of the Endoplasmic Reticulum
Source: PLoS One. 2014 Jan 14;9(1):e85206. doi: 10.1371/journal.pone.0085206 (PMC3891740; doi:10.1371/journal.pone.0085206)

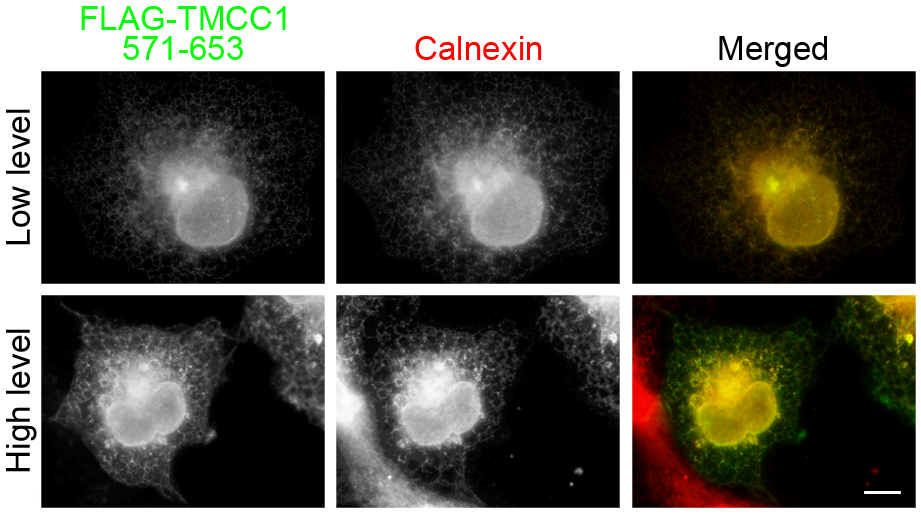

Supplement: Figure S1 — ER defects induced by overexpression of TMCC1 transmembrane domains. COS-7 cells were transfected with a plasmid encoding FLAG-tagged TMCC1(571–653); 24 h post-transfection, cells with low and high levels of exogenous proteins were fixed with methanol and co-stained with FLAG and calnexin antibodies. Scale bar, 10 µm. (TIF) [file pone.0085206.s001.tif]

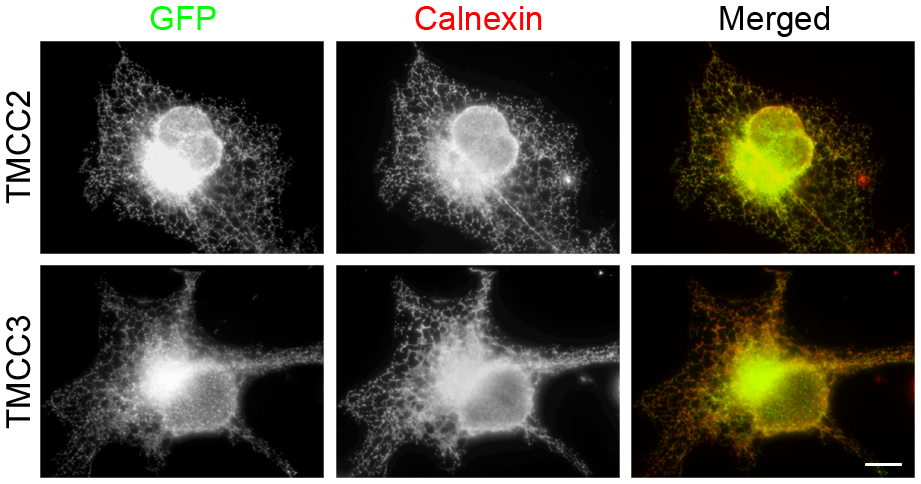

Supplement: Figure S2 — Subcellular localization of TMCC2 and TMCC3. COS-7 cells were transfected with plasmids encoding GFP-tagged TMCC2 or TMCC3; 24 h post-transfection, cells were fixed with methanol and stained with an anti-calnexin antibody. Scale bar, 10 µm. (TIF) [file pone.0085206.s002.tif]
